# Exploring the Therapeutic Potential of *Rehmannia glutinosa*: A Network Pharmacology and Molecular Docking Analysis Across Multiple Diseases

**DOI:** 10.3390/cimb47050329

**Published:** 2025-05-03

**Authors:** Jinyoung Park, Muhammad Yasir, Eun-Taek Han, Jin-Hee Han, Won Sun Park, Jongseon Choe, Wanjoo Chun

**Affiliations:** 1 Department of Pharmacology, Kangwon National University School of Medicine, Chuncheon 24341, Republic of Korea; jinyoung0326@kangwon.ac.kr (J.P.); yasir.khokhar1999@gmail.com (M.Y.); 2 Department of Medical Environmental Biology and Tropical Medicine, Kangwon National University School of Medicine, Chuncheon 24341, Republic of Korea; ethan@kangwon.ac.kr (E.-T.H.); han.han@kangwon.ac.kr (J.-H.H.); 3 Department of Physiology, Kangwon National University School of Medicine, Chuncheon 24341, Republic of Korea; parkws@kangwon.ac.kr; 4 Department of Microbiology and Immunology, Kangwon National University School of Medicine, Chuncheon 24341, Republic of Korea; jchoe@kangwon.ac.kr

**Keywords:** *Rehmannia glutinosa* (Orobanchaceae), network pharmacology, molecular docking, bioactive compounds, protein–protein interaction, allergy, anemia, diabetes, menopause

## Abstract

*Rehmannia glutinosa* (RG), a fundamental herb in traditional Chinese medicine belonging to the Orobanchaceae family, has been widely used for centuries due to its diverse therapeutic properties, including promoting blood circulation, enhancing immunity, managing diabetes, reducing inflammation, and supporting kidney function. Despite its traditional significance, scientific studies on RG’s therapeutic mechanisms remain limited, and its underlying pharmacological pathways are not extensively elucidated. This study employed network pharmacology and molecular docking to identify RG’s active compounds and investigate their therapeutic potential in allergy, anemia, diabetes, and menopause. From an initial pool of 122 compounds, 50 bioactive compounds were screened based on bioavailability and drug-likeness, resulting in 40 active compounds and 11 target proteins closely associated with these conditions. Key active compounds identified included iridoid glycosides (rehmaglutin A, B, C, D, jioglutin A, B, C, jioglutolide) and other bioactive molecules such as caffeic acid, geraniol, 5-hydroxytryptamine, melatonin, and rhodioloside. Molecular docking technology was employed to verify the stable binding of target proteins with active compounds. Protein–protein interaction (PPI) analysis revealed that RG’s core target proteins are central to pathways regulating inflammation, cell survival, apoptosis, and immune response. Enrichment analyses demonstrated that RG’s target proteins intersect significantly with pathways including the AGE-RAGE signaling pathway in diabetic complications, IL-17, HIF-1 signaling, and neuroactive ligand-receptor interactions, all of which are essential in managing diabetes and menopause symptoms. These findings underscore RG’s multi-target therapeutic potential, particularly in modulating immunity, metabolism, and inflammation. This study highlights RG’s potential as a therapeutic agent and provides a framework for future research to further elucidate its mechanisms and support the development of targeted drugs based on RG’s active compounds.

## 1. Introduction

*Rehmannia glutinosa* (Orobanchaceae, RG) is one of the fundamental herbs in traditional Chinese medicine (TCM) and has been utilized for thousands of years due to its diverse pharmacological effects, including improving blood circulation, enhancing immune function, exhibiting anti-inflammatory properties, managing diabetes, and supporting kidney function [1,2]. RG engages with various physiological pathways and contains numerous active compounds, making it effective for treating complex diseases. These pharmacological properties have established RG as an important therapeutic agent for conditions such as allergies, anemia, diabetes, and menopause-related symptoms [3].

Despite the significant role RG plays in traditional medicine, there are notable limitations and challenges in its application. The traditional use of RG relies on empirical knowledge and formulations, often without a clear understanding of specific mechanisms or bioactive targets [4]. Additionally, traditional formulations may suffer from issues such as variability in potency, potential for adverse effects, and lack of standardization, which limits their acceptance in modern evidence-based medicine and the development of RG-based therapeutic drugs [5]. These limitations have restricted RG’s application in contemporary medical practices, underscoring the need for scientific validation and mechanistic elucidation [4].

Network pharmacology has emerged as a powerful approach to bridge these limitations, providing a systematic framework for studying traditional medicines. By integrating bioinformatics and molecular biology, network pharmacology can analyze the interactions between bioactive compounds, target proteins, and disease pathways, offering insights into the complex, multi-target nature of herbal medicines like RG. This approach enables network pharmacology to identify the molecular interactions that underlie therapeutic effects, but also helps optimize and standardize herbal formulations for clinical use. [6].

This research employed network pharmacology to address the limitations of RG by identifying its active compounds and target proteins, thereby elucidating its therapeutic mechanisms. By focusing on conditions such as allergy, anemia, diabetes, and menopause, we aim to provide a comprehensive understanding of RG’s multi-target actions and its effects on key biological pathways, such as inflammation, apoptosis, and immune regulation. This multi-disease analysis reveals RG’s broad therapeutic potential and underscores its role as a multi-target agent, a concept less explored in earlier studies. Through protein–protein interaction (PPI) network analysis, Gene Ontology (GO) enrichment, and Kyoto Encyclopedia of Genes and Genomes (KEGG) pathway analysis, our study reveals the complex interactions and pharmacological pathways involved in RG’s effects. The potential interaction of target proteins with active compounds was identified by molecular docking.

The findings from this research underscore the potential of RG in advancing the development of targeted therapeutic drugs. By decoding the mechanisms through which RG exerts its therapeutic effects, this study contributes to the integration of TCM into modern pharmacology, potentially paving the way for the development of more effective, multi-targeted treatments derived from traditional herbs. This work provides a valuable framework for future research, demonstrating how network pharmacology can enhance our understanding of TCM and contribute to the development of standardized, evidence-based therapies.

**Figure 1:**
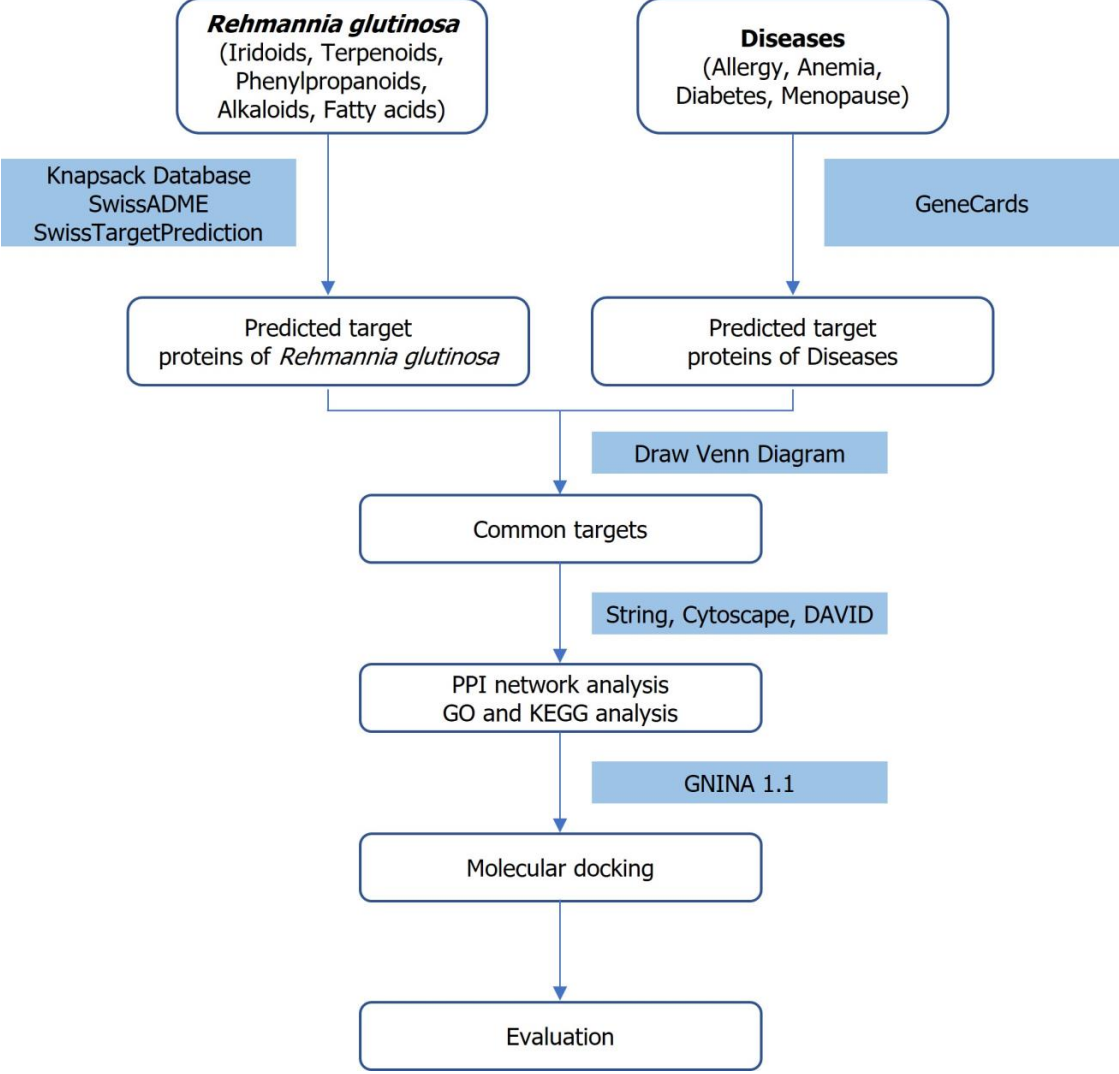
Workflow integrating network pharmacology and molecular docking to investigate the therapeutic potential of *Rehmannia glutinosa* (Orobanchaceae).

The overall research strategy-integrating network pharmacology, protein-protein interaction analysis, pathway enrichment and molecular docking to explore the therapeutic potential of *Rehmannia glutinosa*-is schematically illustrated Figure 1.

## 2. Materials and Methods

### 2.1. Mining of Active Compounds and Targets in Rehmannia glutinosa

Using KNApSAcK family databases (http://kanaya.naist.jp/KNApSAcK_Family) and SwissADME (http://www.swissadme.ch), active compounds of *Rehmannia glutinosa* (bioavailability ≥ 0.3, drug-likeness≥ 0.18) were screened to predict target proteins corresponding to these active compounds. The targets of the RG were predicted using the Swiss Target Prediction webserver (http://www.swisstargetprediction.ch/). The targets related to diseases were obtained from the GeneCards database (https://www.genecards.org/). When searching for targets, the terms allergy, anemia, diabetes, menopause, and *Homo sapiens* were used. The target names were standardized into official gene symbols using the UniProt database (https://www.uniprot.org/). Using Draw Venn Diagram (https://bioinformatics.psb.ugent.be/webtools/Venn/), Venn diagrams of disease target proteins and active compound target proteins of RG were obtained.

### 2.2. Protein–Protein Interaction Network Construction and Analysis

The common targets between RG and allergy, anemia, diabetes, and menopause were imported to the STRING 12.0 database (https://string-db.org/) to construct a protein–protein interaction (PPI) network. The organism used was *Homo sapiens*, the minimum required interaction score was set to ‘high confidence > 0.7’, and other parameters were kept at their default settings. Cytoscape 3.10.1 was used for visualization, and the plug-in cytoHubba was executed to calculate the ranking of common target proteins. We selected the top 10 proteins using the algorithms MCC and Degree in cytoHubba.

### 2.3. GO and KEEG Enrichment Analysis

The common proteins between RG and the diseases, allergy, anemia, diabetes, and menopause, were introduced into the DAVID bioinformatics resources (https://david.ncifcrf.gov/home.jsp) to analyze Gene Ontology (GO) annotation and Kyoto Encyclopedia of Genes and Genomes (KEGG) pathways. The selected identification code was ‘OFFICIAL_GENE_SYMBOL’ and the species was specified as *Homo sapiens* [7,8]. SRPLOT (https://www.bioinformatics.com.cn/en) was used to visualize the results. To visualize the results of the GO analysis, the top 10 *p*-values for Biological Processes (BP), Molecular Functions (MF), and Cellular Components (CC) were selected. For the KEGG pathway analysis, the top 10 pathways based on *p*-values were chosen. The *p*-value was adjusted to 0.05 or less. The compound-target-pathway network elucidated the pathways and roles of the target proteins.

### 2.4. Construction of the Active Compound-Target-Disease Network

The network construction was visualized using a Sankey diagram, which can intuitively show the associations between the active compounds of RG, their targets, and the four respective diseases. The Sankey diagram was drawn using Sankey MATIC (https://sankeymatic.com/build/).

### 2.5. Molecular Docking

Molecular docking was performed to confirm the interactions between the active compounds and the targets of RG in disease treatment and to verify the accuracy of the network pharmacology prediction. The SDF structures of the active compounds were obtained from the PubChem database (https://pubchem.ncbi.nlm.nih.gov). The 3D structure of the target protein was obtained from the Protein Data Bank (PDB) database (https://www.rcsb.org/) and preprocessed as necessary. Molecular docking simulations were conducted using GNINA 1.1, a recently developed software that utilizes deep convolutional neural networks as a scoring function to evaluate protein-ligand interaction [9]. The docking process was performed by specifying the binding site of the PDB structure for each target protein. For target proteins that were not complexed with an inhibitor, such as 1ALU and 3LTQ, GNINA’s AutoBox feature was applied to automatically determine the docking box. GNINA 1.1 outperforms conventional empirical scoring methods such as AutoDock Vina in virtual screening tasks, demonstrating improved early enrichment factors and enhanced pose prediction accuracy [9,10]. Each docking experiment was independently performed three times using different random seeds in GNINA to ensure the reproducibility and reliability of the binding predictions. The GNINA 1.1 score represents the predicted binding affinity between the active compound and the target protein, where a lower score indicates a stronger binding potential.

## 3. Results

### 3.1. Collection of Active Compounds in RG and Target Proteins of Diseases

**Figure 2:**
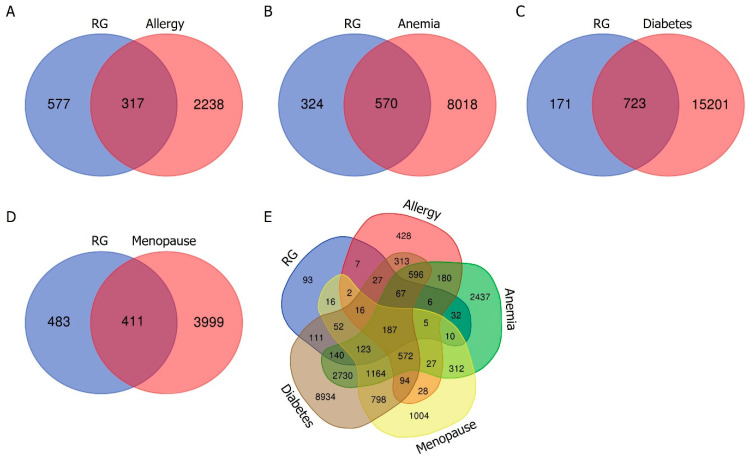
Venn diagrams illustrating the overlap of target proteins between RG and allergy (**A**), RG and anemia (**B**), RG and diabetes (**C**), and RG and menopause (**D**), respectively. The blue section represents the target proteins of RG, while the red section represents the target proteins of each disease. (**E**) Venn diagram illustrating the combination of target proteins for RG, allergy, anemia, diabetes, and menopause.

The active compounds of RG were mined by the KNApSAcK family databases. As for the active compounds, a total of 50 compounds were selected based on bioavailability and drug-likeness. Next, we used the SwissTargetPrediction database to predict potential target proteins associated with these active compounds. This comprehensive analysis yielded 894 targets specific to RG that were established. In parallel, we obtained lists of target proteins associated with allergy, anemia, diabetes, and menopause through independent means from the GeneCards database. We obtained 2555 targets for allergy, 8588 targets for anemia, 15,924 targets for diabetes, and 4410 targets for menopause, respectively. To identify potential therapeutic targets at the intersection of RG’s bioactive compounds and each disease, a Venn diagram was generated. This analysis revealed overlapping proteins between the target proteins associated with RG’s bioactive compounds and each disease: 317 for allergy, 570 for anemia, 723 for diabetes, and 411 for menopause. Ultimately, 187 common target proteins were identified across the four diseases and RG (Figure 2).

### 3.2. Construction of Protein–Protein Interaction Network for Disease and RG

**Figure 3:**
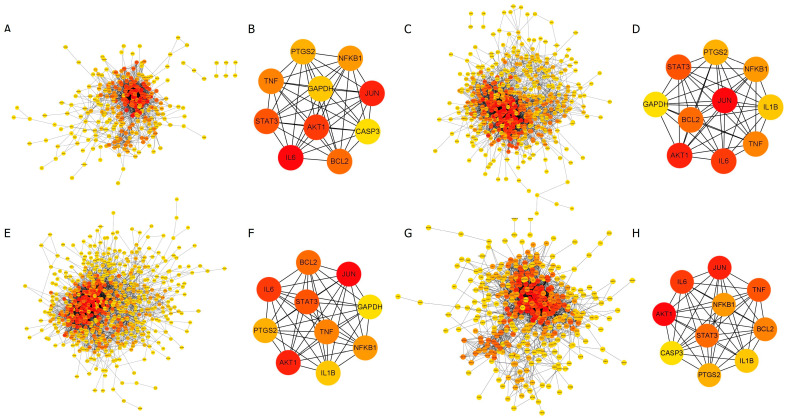
Protein–protein interaction (PPI) network analysis. The intensity of the red color in the nodes represents the strength of the interaction between proteins. (**A**,**B**) RG and allergy, (**C**,**D**) RG and anemia, (**E**,**F**) RG and diabetes, as well as (**G**,**H**) RG and menopause. (**B**,**D**,**F**,**H**) The core PPI network of the disease-specific top 10 target proteins is selected from each common target protein.

The common target proteins between RG and each disease were imported into Cytoscape 3.10.1 to construct a protein–protein interaction (PPI) network. For allergy, 317 common target proteins were identified, of which 32 non-interacting proteins were excluded, resulting in a network consisting of 285 nodes and 1846 edges (Figure 3). In this network, nodes represent proteins, and edges denote interactions between these proteins. The color gradient of the nodes reflects the level of interaction, with higher interaction levels indicated by redder colors.

For anemia, out of 570 common target proteins, 40 were excluded, resulting in a network with 530 nodes and 3858 edges. The PPI network for diabetes consisted of 680 nodes and 4609 edges, while for menopause, the network included 387 nodes and 2893 edges (Figure 3).

The top 10 target proteins for each disease were identified using the MCC algorithm in the CytoHubba plugin, revealing highly similar results across diseases. (Table 1). Analysis of the PPI network of 187 common target proteins shared between RG and the four diseases produced a consolidated network comprising 174 nodes and 1347 edges. The top 10 target proteins within this network were JUN, AKT1, IL6 (Interleukin 6), TNF (Tumor necrosis factor), STAT3 (Signal transducer and activator of transcription 3), BCL2 (B-cell lymphoma 2), NFKB1 (Nuclear factor kappa-B), PTGS2 (Prostaglandin-endoperoxide synthase 2), IL1B (Interleukin-1 beta), and CASP3 (Caspase 3). The experimental data were provided in the Supplementary Data (Figure S1, Table S1).

### 3.3. GO and KEGG Pathway Enrichment Analysis

To elucidate the biological significance of the identified target proteins, GO enrichment analysis was conducted using the DAVID bioinformatics resources. The analysis included 317 common proteins related to allergy, 570 for anemia, 723 for diabetes, and 411 for menopause. The GO enrichment analysis yielded 1221 GO terms for allergy, 1698 for anemia, 1808 for diabetes, and 1585 for menopause, each with a *p*-value < 0.05. These terms were categorized into biological process (BP), cellular component (CC), and molecular function (MF).

**Figure 4:**
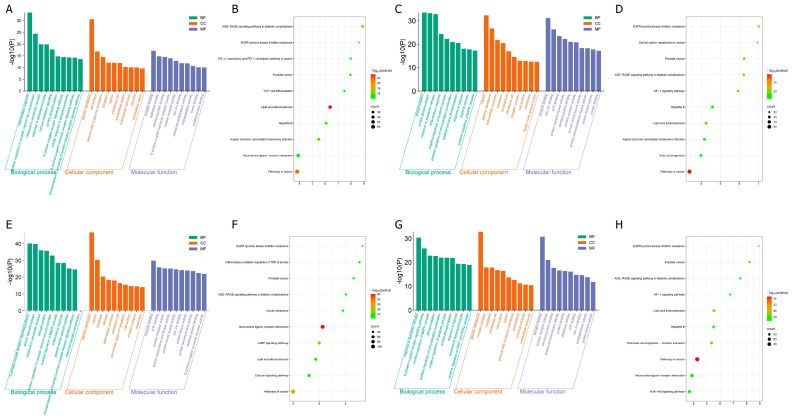
Functional enrichment of the target proteins: (**A**,**C**,**E**,**F**) GO enrichment annotation analysis. The top 10 terms in each GO category are presented as bar charts, where BP, CC, and MF are represented in green, orange, and slate blue, respectively. (**B**,**D**,**F**,**H**) KEGG pathway enrichment analysis of target proteins. (**A**,**B**) RG and allergy, (**C**,**D**) RG and anemia, (**E**,**F**) RG and diabetes, (**G**,**H**) RG and menopause.

For allergy, the BP terms included inflammatory response, positive regulation of cytosolic calcium ion concentration, and response to xenobiotic stimulus. The CC terms included plasma membrane, cell surface, and the external side of the plasma membrane. The MF terms included enzyme binding, peptidase activity, and identical protein binding. The top 10 GO terms are shown in Figure 4A.

For anemia, the BP terms included phosphorylation, protein phosphorylation, and response to xenobiotic stimulus. The CC terms included cytosol, plasma membrane, and extracellular exosome. The MF terms included enzyme binding, zinc ion binding, and ATP binding. The top 10 GO terms are shown in Figure 4C.

For diabetes, the BP terms included G protein-coupled receptor signaling pathway, protein phosphorylation, and response to xenobiotic stimulus. The CC terms included plasma membrane, cytosol, and cytoplasm. The MF terms included enzyme binding, ATP binding, and nuclear receptor activity. The top 10 GO terms are shown in Figure 4E.

For menopause, the BP terms include response to xenobiotic stimulus, G-protein-coupled receptor signaling pathway, positive regulation of MAPK cascade, CC includes plasma membrane, receptor complex, and cytoplasm, MF includes enzyme binding, nuclear receptor activity, and identical protein binding. The top 10 GO terms are shown in Figure 4G.

Through KEGG enrichment analysis, 181 pathways were identified for allergy, 196 for anemia, 201 for diabetes, and 182 for menopause. The top 10 pathways for each condition are presented in Figure 4B,D,F,H.

For allergy, the main pathways included AGE-RAGE signaling pathway in diabetic complications, EGFR tyrosine kinase inhibitor resistance, PD-L1 expression and PD-1 checkpoint pathway in cancer, prostate cancer, TH17 cell differentiation, lipid and atherosclerosis, hepatitis B, Kaposi sarcoma-associated herpesvirus infection, neuroactive ligand-receptor interaction, and pathways in cancer (Figure 4B).

For anemia, the main pathways included pathways in cancer, lipid, and atherosclerosis, AGE-RAGE signaling pathway in diabetic complications, EGFR tyrosine kinase inhibitor resistance, HIF-1 signaling pathway, prostate cancer, hepatitis B, Kaposi sarcoma-associated herpesvirus infection, central carbon metabolism in cancer, and viral carcinogenesis (Figure 4D).

For diabetes, the main pathways included neuroactive ligand-receptor interaction, pathways in cancer, cAMP signaling pathway, calcium signaling pathway, lipid and atherosclerosis, inflammatory mediator regulation of TRP channels, prostate cancer, insulin resistance, AGE-RAGE signaling pathway in diabetic complications, and EGFR tyrosine kinase inhibitor resistance (Figure 4F).

For menopause, the main pathways included pathways in cancer, lipid and atherosclerosis, chemical carcinogenesis-receptor activation, prostate cancer, EGFR tyrosine kinase inhibitor resistance, neuroactive ligand-receptor interaction, AGE-RAGE signaling pathway in diabetic complications, hepatitis B, PI3K-Akt signaling pathway, and HIF-1 signaling pathway (Figure 4H).

### 3.4. Construction of the Active Compound-Core Target Protein-Disease Network

**Figure 5:**
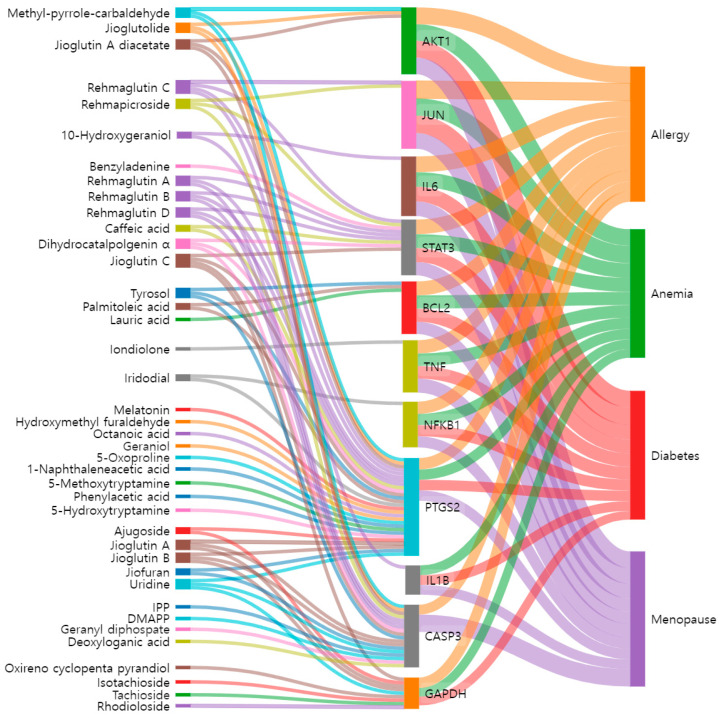
The Sankey diagram reveals the relationship between active compounds of RG, core targets, and diseases. The left blocks represent the active compounds, the middle blocks represent the core targets, and the right blocks represent the diseases.

The Sankey diagram was used to visually express the association and flow between the active compounds in RG, their target proteins, and four related diseases. We found 40 active compounds targeting the top 11 proteins that control each disease (Figure 5). This network clearly shows which target proteins the active compounds act on in each disease. The mechanisms by which many of these active compounds interact with the target proteins have already been documented (Table 2). Therefore, the active compounds of RG, including geraniol, caffeic acid, melatonin, and iridodial, were used in subsequent molecular docking studies. Nodes represent active compounds, target proteins, and diseases, while edges, depicted as lines connecting the nodes, illustrate the flow of relationships between them.

### 3.5. Molecular Docking of Active Compounds and Core Target Proteins

Molecular docking simulations were performed to evaluate the binding interactions between the top 11 core target proteins and 40 active compounds. The selected target proteins included AKT1(PDB ID: 3MV5), JUN(PDB ID: 4Y46), IL6(PDB ID: 1ALU), STAT3(PDB ID: 6NJS), BCL2(PDB ID: 2W3L), TNF(PDB ID: 2AZ5), NFKB1(PDB ID: 8TQD), PTGS2(PDB ID: 5F19), IL1B(PDB ID: 3LTQ), CASP3(PDB ID: 3EDQ), and GAPDH(PDB ID: 6IQ6). For target proteins co-crystallized with inhibitors, molecular docking was conducted using the binding sites of the co-crystallized ligands. However, for 1ALU (IL6) and 3LTQ (IL1B), which were not complexed with inhibitors, the docking grid was defined using AutoBox, an automated approach in GNINA that identifies potential binding sites based on the structural features of the protein. AutoBox refines the docking search to biologically relevant pockets, increasing computational efficiency and improving the accuracy of docking predictions. This approach enhances the reliability of docking results, particularly in cases where no co-crystallized ligand is available. To ensure accuracy and reproducibility, each docking experiment was performed three times through independent docking simulations (Table S2). The resulting binding scores were averaged, and the standard deviation was calculated to assess variability (Table 3). The docking scores ranged from −3.37 to −9.04 kcal/mol. Since binding energy values lower than 0 kcal/mol indicate spontaneous interactions between the compound and the target protein, the negative binding affinities suggest favorable compound binding. Moreover, a larger absolute binding energy value correlates with a stronger binding affinity.

## 4. Discussion

This study aimed to screen active ingredients in *Rehmannia glutinosa* (Orobanchaceae, RG) and identify potential therapeutic targets for diseases, uncovering mechanisms for treating conditions such as allergies, anemia, diabetes, and menopause. From an initial pool of 122 constituents, 50 bioactive compounds were screened based on bioavailability (≥0.3) and drug-likeness (≥0.18). The identified active compounds include iridoid glycosides, such as rehmaglutin A, B, C, and D, jioglutin A, B, C, and jioglutolide, as well as other bioactive compounds like caffeic acid, geraniol, 5-hydroxytryptamine, melatonin, and rhodioloside. Previous studies indicate that these constituents have antioxidant, anti-inflammatory, anti-cancer properties, and can improve blood circulation [3,15,19,39].

PPI (protein–protein interaction) analysis is essential for understanding the complex relationships among multiple proteins involved in disease pathogenesis and identifying key targets for drug action. It plays a crucial role in pinpointing core proteins that significantly contribute to disease progression. Therefore, we conducted a PPI analysis to identify the top 10 target proteins shared between the disease and RG, which are likely to serve as critical therapeutic targets. Among them, eight target proteins—AKT1, JUN, IL6, STAT3, BCL2, TNF, NFKB1, and PTGS2-matched perfectly, while the remaining three proteins, IL1B, CASP3, and GAPDH, showed differences. This result highlights the significant overlap in the target proteins involved in the pathophysiology of allergy, anemia, diabetes, and menopause, and the pharmacological action of RG. The identification of these eight common targets highlights their central role in the shared molecular mechanisms underlying these diseases and RG’s therapeutic effects.

For instance, AKT1 and JUN are essential in modulating cellular responses to oxidative stress and inflammatory stimuli, both of which are crucial for managing allergic reactions and maintaining immune balance [40]. Similarly, the STAT3 and BCL2 pathways—known for their roles in apoptosis and immune response—are modulated by compounds such as caffeic acid and melatonin, potentially explaining RG’s impact on allergy and anemia by promoting cell survival and reducing inflammation [41,42]. Furthermore, IL6, IL1B, and TNF are pro-inflammatory cytokines, which are associated with insulin resistance and the development of chronic inflammation [1,39]. Elevated IL6 and TNF levels have been observed in patients with type 2 diabetes, and these cytokines are linked to disease progression through pathways such as AGE-RAGE signaling pathways associated with diabetes complications [43,44].

GO and KEGG analyses revealed that the target proteins of RG are involved in critical pathways associated with diabetic complications, including the AGE-RAGE signaling pathway, IL-17 and HIF-1 signaling pathways, and neuroactive ligand-receptor interactions. These pathways are also closely linked to managing menopause-related symptoms [45]. Rhodioloside and 5-hydroxytryptamine may alleviate diabetes by modulating oxidative stress and inflammatory responses [46,47,48]. Palmitoleic acid, geraniol, and tyrosol play crucial roles in anti-inflammatory and anti-cancer processes through their regulation of PTGS2 (Prostaglandin-Endoperoxide Synthase 2) [23]. PTGS2, also known as COX-2 (Cyclooxygenase-2), is intimately involved in bone metabolism and has significant implications for osteoporosis in postmenopausal women [49].

Tyrosol plays a key role in inhibiting apoptosis in cells by regulating BCL2 and CASP3 [17]. GAPDH is a key player in the mechanisms underlying the onset and progression of diabetic retinopathy [50,51], suggesting that active compounds targeting GAPDH could hold significant therapeutic potential. The presence of three distinct target proteins (IL1B, CASP3, GAPDH) for certain diseases indicates that RG influences shared pathways while also exhibiting disease-specific mechanisms of action. The overlap in target proteins supports the idea that RG may be effective as a multi-disease therapy, particularly for conditions with inflammatory or immune-related components.

The active compounds identified through network pharmacology were further evaluated through molecular docking to confirm their potential interactions with target proteins. The results demonstrated stable binding affinities. To support our findings, we conducted a literature review and included references only to compounds previously reported to interact with the respective target proteins. This approach reinforces the validity of our predictions and highlights the potential pharmacological relevance of the identified compounds.

While earlier studies highlighted catalpol and rehmanniosides in diabetes [52] or ovarian dysfunction [53], our findings expand the repertoire of RG’s active compounds. The active compounds of RG analyzed in this study may be present at low concentrations or occur in higher abundance in other plant species. However, our focus lies in the therapeutic effects that arise from the synergistic action of multiple components of RG on multiple targets. This is one of the key strengths of network pharmacology, which, in contrast to classical pharmacology that emphasizes single compounds and single targets, adopts a systems–level perspective. Our study demonstrates that multiple active compounds in RG can act cooperatively across various disease pathways to exert integrated therapeutic effects.

Furthermore, although in silico predictions are inherently limited by the accuracy of database information and the static nature of molecular docking, they serve as a valuable hypothesis-generating platform that can inform future experimental research. Therefore, despite these limitations, our predictions remain biologically plausible, and future in vitro and in vivo studies are warranted to validate the pharmacokinetics and therapeutic efficacy of the identified compounds.

This study confirms the potential applicability of RG in patients with allergies, anemia, diabetes, and menopause, as well as in those with complex comorbidities, providing a basis for the development of personalized treatment strategies. Furthermore, molecular docking and network pharmacology analysis demonstrated the clinical relevance of RG by elucidating the interactions between its active compounds and target proteins, indicating its potential for therapeutic applications.

## Figures and Tables

**Table 1:** Disease-specific top 10 target proteins.

	Allergy	Anemia	Diabetes	Menopause
1	IL6	JUN	JUN	AKT1
2	JUN	AKT1	AKT1	JUN
3	AKT1	IL6	IL6	IL6
4	STAT3	STAT3	STAT3	TNF
5	BCL2	BCL2	BCL2	STAT3
6	TNF	TNF	TNF	BCL2
7	NFKB1	NFKB1	NFKB1	NFKB1
8	PTGS2	PTGS2	PTGS2	PTGS2
9	GAPDH	IL1B	IL1B	IL1B
10	CASP3	GAPDH	GAPDH	CASP3

**Table 2:** Compounds predicted as active through network pharmacology analysis, with those supported by literature denoted by citations.

Target Protein	Compounds
AKT1	Jioglutolide, Jioglutin A diacetate, Methyl-pyrrole-carbaldehyde
JUN	Rehmaglutin C, Rehmapicroside
IL6	10-Hydroxygeraniol [11,12]
STAT3	Benzyladenine, Caffeic acid [13,14,15,16], Rehmaglutin A, Jioglutin C, Rehmaglutin D, Rehmaglutin B, Rehmaglutin C, Rehmapicroside, Dihydrocatalpolgenin α
BCL2	Lauric acid, Palmitoleic acid, Tyrosol [17]
TNF	Iondiolone
NFKB1	Iridodial [18]
PTGS2	Caffeic acid [19], Phenylacetic acid, Geraniol [20,21,22], Octanoic acid, Palmitoleic acid [23], 5-Hydroxytryptamine [24,25], Iridodial, 5-Oxoproline, Jiofuran, Jioglutolide, Ajugoside [26], Rehmaglutin A, Jioglutin C, Rehmaglutin D, Rehmaglutin B, Jioglutin A, Jioglutin B, Rehmaglutin C, Tyrosol [27,28,29,30], Hydroxymethyl furaldehyde [31], 5-Methoxytryptamine, Melatonin [32,33,34], 10-Hydroxygeraniol [35], Jioglutin A diacetate, Dihydrocatalpolgenin α, Methyl-pyrrole-carbaldehyde, 1-Naphthaleneacetic acid
IL1B	Rehmaglutin C
CASP3	Geranyl diphosphate, Deoxyloganic acid, Jiofuran, Jioglutolide, Rehmaglutin A, Jioglutin C, Rehmaglutin D, Rehmaglutin B, Jioglutin A, Jioglutin B, Rehmapicroside, Uridine [36,37,38], Tyrosol [17], DMAPP, IPP, Jioglutin A diacetate, Dihydrocatalpolgenin α, Methyl-pyrrole-carbaldehyde
GAPDH	Ajugoside, Jioglutin C, Jioglutin A, Jioglutin B, Tachioside, Isotachioside, Uridine, Rhodioloside, Oxireno cyclopenta pyrandiol

Footnote: The compounds denoted with literature references were previously reported to be active, while those without literature references were identified as potentially active through the network pharmacology analysis performed in this study.

**Table 3:** The molecular docking energy between active compounds and core targets.

**Target Protein**	**Compounds**	**Binding** **Energy (kcal/mol)**	Target protein	Compounds	Binding Energy (kcal/mol)	Target Protein	Compounds	Binding Energy (kcal/mol)	Target Protein	Compounds	Binding Energy (kcal/mol)
AKT1	Inhibitor 1 *	−6.51 ± 0.59	TNF	Inhibitor 5 *	−8.91 ± 0.58	PTGS2	Rehmaglutin C	−5.34 ± 0.30	CASP3	Rehmapicroside	−5.79 ± 0.61
	Jioglutolide	−5.81 ± 0.43		Iondiolone	−6.34 ± 0.69		Tyrosol	−5.39 ± 0.70		Uridine	−4.79 ± 0.54
	Jioglutin A diacetate	−5.40 ± 1.48	NFKB1	Inhibitor 6 *	−4.12 ± 0.62		Hydroxymethyl furaldehyde	−4.41 ± 0.58		Tyrosol	−3.43 ± 0.41
	Methyl-pyrrole- carbaldehyde	−4.61 ± 0.26		Iridodial	−3.51 ± 0.55		5-Methoxytryptamine	−5.26 ± 0.47		DMAPP	−3.83 ± 0.39
JUN	Inhibitor 2 *	−8.57 ± 0.86	PTGS2	Inhibitor 7 *	−7.27 ± 1.04		Melatonin	−6.36 ± 0.47		IPP	−4.07 ± 0.46
	Rehmaglutin C	−4.42 ± 0.27		Caffeic acid	−6.29 ± 0.76		10-Hydroxygeraniol	−5.06 ± 0.71		Jioglutin A diacetate	−4.57 ± 0.84
	Rehmapicroside	−7.18 ± 0.78		Phenylacetic acid	−5.00 ± 0.96		Jioglutin A diacetate	−6.48 ± 0.54		Dihydrocatalpolgenin α	−4.62 ± 0.45
IL6	10-Hydroxygeraniol	−4.10 ± 0.58		Geraniol	−4.94 ± 0.86		Dihydrocatalpolgenin α	−5.48 ± 0.18		Methyl-pyrrole-carbaldehyde	−3.37 ± 0.63
STAT	Inhibitor 3 *	−7.93 ± 0.67		Octanoic acid	−4.34 ± 0.67		Methyl-pyrrole-carbaldehyde	−4.31 ± 0.69	GAPDH	Inhibitor 9 *	−4.15 ± 0.37
	Benzyladenine	−4.42 ± 0.44		Palmitoleic acid	−5.14 ± 0.36		1-Naphthaleneacetic acid	−5.95 ± 0.38		Ajugoside	−9.04 ± 0.81
	Caffeic acid	−4.65 ± 0.55		5-Hydroxytryptamine	−5.44 ± 0.49	IL1B	Rehmaglutin C	−4.01 ± 0.38		Jioglutin C	−7.18 ±0.47
	Rehmaglutin A	−4.26 ± 0.23		Iridodial	−5.19 ± 0.30	CASP3	Inhibitor 8 *	−3.57 ± 0.77		Jioglutin A	−7.28 ± 0.47
	Jioglutin C	−4.54 ± 0.41		5-Oxoproline	−5.25 ± 0.78		Geranyl diphosphate	−4.45 ±0.47		Jioglutin B	−7.28 ± 0.47
	Rehmaglutin D	−4.39 ± 0.46		Jiofuran	−5.27 ± 0.54		Deoxyloganic acid	−6.00 ±0.80		Tachioside	−8.36 ± 0.47
	Rehmaglutin B	−4.69 ± 0.48		Jioglutolide	−5.95 ± 0.88		Jiofuran	−4.23 ± 0.50		Isotachioside	−8.34 ± 0.37
	Rehmaglutin C	−4.15 ± 0.33		Ajugoside	−8.37 ± 0.42		Jioglutolide	−4.18 ± 0.63		Uridine	−7.14 ±0.48
	Rehmapicroside	−5.66 ± 0.53		Rehmaglutin A	−5.93 ± 0.32		Rehmaglutin A	−4.52 ± 0.49		Rhodioloside	−7.93 ± 0.64
	Dihydrocatalpolgenin α	−4.34 ± 0.33		Jioglutin C	−6.23 ± 0.26		Jioglutin C	−4.78 ± 0.52		Oxireno cyclopenta pyrandiol	−6.65 ± 0.52
BCL2	Inhibitor 4 *	−9.19 ± 0.4		Rehmaglutin D	−5.53 ± 0.32		Rehmaglutin D	−4.36 ± 0.48			
	Lauric acid	−4.34 ± 0.33		Rehmaglutin B	−5.75 ± 0.42		Rehmaglutin B	−4.57 ± 0.50			
	Palmitoleic acid	−4.71 ± 0.44		Jioglutin A	−5.92 ± 0.26		Jioglutin A	−4.50 ± 0.59			
	Tyrosol	−4.21 ± 0.35		Jioglutin B	−5.81 ± 0.39		Jioglutin B	−4.41 ± 0.52			

* Indicates known inhibitors of each target protein. Inhibitor 1: (3R)-1-(5-methyl-7H-pyrrolo[2,3-d]pyrimidin-4-yl)pyrrolidin-3-amine (CHEMBL1173273). Inhibitor 2: 1-{trans-4-[(8-cyclopentyl-7-oxo-7,8-dihydropyrido[2,3-d]pyrimidin-2-yl)amino]cyclohexyl}-3-propan-2-ylurea (CHEMBL3577877). Inhibitor 3: [(2-{[(5S,8S,10aR)-3-acetyl-8-({(2S)-5-amino-1-[(diphenylmethyl)amino]-1,5-dioxopentan-2-yl}carbamoyl)-6-oxodecahydropyrrolo[1,2-a][1,5]diazocin-5-yl]carbamoyl}-1H-indol-5-yl)(difluoro)methyl]phosphonic acid (PubChem SID 404647587). Inhibitor 4: 1-(2-{[(3S)-3-(aminomethyl)-3,4-dihydroisoquinolin-2(1H)-yl]carbonyl}phenyl)-4-chloro-5-methyl-N,N-diphenyl-1H-pyrazole-3-carboxamide (CHEMBL503454). Inhibitor 5: 6,7-dinethyl-3-[(methyl{2-[ methyl ({1-[3-(trifluoromethyl(phenyl]-1H-indol-3-yl}methyl)amino]ethyl}amino)methyl]-4H-chromen-4-one (CHEMBL255489). Inhibitor 6: 1-(2-bromo-4-chlorophenyl)-N-{(3S)-1-[(E)-iminomethyl]pyrrolidin-3-yl}methanesulfonamide (PubChem CID 171362236). Inhibitor 7: Protoporphyrin IX (CHEMBL1325592). Inhibitor 8: Aspartic aldehyde (PubChem CID 6540254). Inhibitor 9: Monomethyl maleate (PubChem CID 5354456).

## Data Availability

The data that support the findings of this study are available from the corresponding author upon reasonable request.

## Supplementary Materials

The following supporting information can be downloaded at https://www.mdpi.com/article/10.3390/cimb47050329/s1.
