# Supplementary material for: Exploring the Therapeutic Potential of Rehmannia glutinosa: A Network Pharmacology and Molecular Docking Analysis Across Multiple Diseases
Source: Curr Issues Mol Biol. 2025 May 3;47(5):329. doi: 10.3390/cimb47050329 (PMC12110064; doi:10.3390/cimb47050329)
Supplement: Supplementary file 1 [file cimb-47-00329-s001.zip › cimb-3588450-supplementary.pdf]

Network Pharmacology and Molecular Docking Analysis of *Rehmannia glutinosa*: Insights into its Therapeutic Mechanisms Across Multiple Diseases

Jinyoung Park[1], Muhammad Yasir[1], Eun-Taek Han[2], Jin-Hee Han[2],

Won Sun Park[3], Jongseon Choe[4], Wanjoo Chun[1,*]

[1]Department of Pharmacology, Kangwon National University School of Medicine, Chuncheon, 24341, Republic of Korea;

[2]Department of Medical Environmental Biology and Tropical Medicine, Kangwon National University School of Medicine, Chuncheon, 24341, Republic of Korea;

[3]Department of Physiology, Kangwon National University School of Medicine, Chuncheon, 24341, Republic of Korea;

[4]Department of Microbiology and Immunology, Kangwon National University School of Medicine, Chuncheon, 24341, Republic of Korea

Corresponding author: Dr. Wanjoo Chun, Department of Pharmacology Kangwon National University School of Medicine, Kangwon National University, Email: wchun@kangwon.ac.kr, Phone: +82-33-250-8853.

1. Methods

1.1. Protein-Protein Interaction Network Construction and Analysis

The 187 common targets shared between RG, allergy, anemia, diabetes and menopause were imported into the STRING 12.0 database (http://string-db.org/) to construct a protein-protein interaction (PPI) network. The species was set to *Homo sapiens*, with the minimum required interaction score set at 'high confidence > 0.7', while all other parameters were maintained at their default settings. The resulting network was visualized using Cytoscape software (version 3.10.1). To identify key target proteins, the cytoHubba plug-in was employed, and the top 10 core proteins were selected based on the MCC (Maximal Clique Centrality) and Degree algorithms.

1.2 Molecular Docking

Molecular docking simulations were performed using GNINA software (version 1.1). For each target protein, the binding site was defined based in its PDB structure. To ensure the reproducibility and reliability of the docking result, each docking experiment was independently conducted three times using different random seeds. The molecular docking energies from these three independent simulations were presented as the final results.

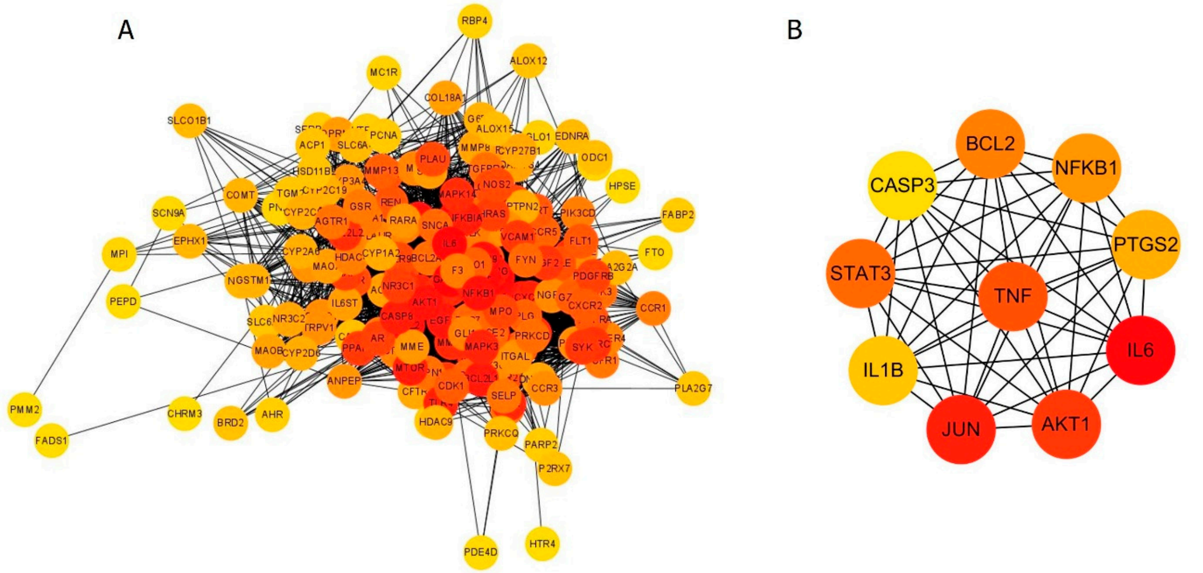

Fig. S1. (A) PPI network analysis of 187 common target proteins of RG, allergy, anemia, diabetes, and menopause. (B) The core PPI network of the top 10 target proteins selected from 187 common target proteins

Table. S1. Comparison of disease-specific top 10 target proteins and the top 10 target proteins shared among RG and the four diseases.

Sr No	**Allergy**	**Anemia**	**Diabetes**	**Menopause**	**Total**
1	IL6	JUN	JUN	AKT1	IL6
2	JUN	AKT1	AKT1	JUN	JUN
3	AKT1	IL6	IL6	IL6	AKT1
4	STAT3	STAT3	STAT3	TNF	TNF
5	BCL2	BCL2	BCL2	STAT3	STAT3
6	TNF	TNF	TNF	BCL2	BCL2
7	NFKB1	NFKB1	NFKB1	NFKB1	NFKB1
8	PTGS2	PTGS2	PTGS2	PTGS2	PTGS2
9	GAPDH	IL1B	IL1B	IL1B	IL1B

Table. S2. Molecular docking energy of compounds with core targets (Triplicate results)

1. AKT1_3mv5

	Jioglutolide			Jioglutin A diacetate			Methyl-pyrrole-carbaldehyde		
	Run 1 Binding energy (kcal/mol)	Run 2 Binding energy (kcal/mol)	Run 3 Binding energy (kcal/mol)	Run 1 Binding energy (kcal/mol)	Run 2 Binding energy (kcal/mol)	Run 3 Binding energy (kcal/mol)	Run 1 Binding energy (kcal/mol)	Run 2 Binding energy (kcal/mol)	Run 3 Binding energy (kcal/mol)
1	-5.52	-6.14	-5.19	-4.26	-4.41	-3.89	-4.69	-5.14	-5.14
2	-6.04	-5.73	-6.14	-4.21	-7.32	-3.79	-5.15	-4.60	-4.69
3	-6.06	-5.17	-5.27	-4.49	-4.01	-3.18	-4.65	-4.48	-4.71
4	-5.31	-5.63	-5.23	-3.79	-7.46	-3.46	-4.30	-4.69	-4.52
5	-5.92	-6.06	-5.28	-4.76	-6.44	-6.99	-4.64	-4.65	-4.65
6	-5.94	-5.97	-5.77	-4.09	-4.36	-6.52	-4.77	-4.14	-4.67
7	-6.66	-5.31	-6.09	-4.36	-6.74	-4.75	-4.21	-4.66	-4.17
8	-5.68	-5.92	-6.65	-7.33	-6.65	-7.28	-4.52	-4.24	-4.53
9	-5.89	-6.65	-5.68	-7.05	-6.68	-7.47	-4.48	-4.48	-4.80

2. JUN_4y46

	Rehmaglutin C			Rehmapicroside		
	Run 1 Binding energy (kcal/mol)	Run 2 Binding energy (kcal/mol)	Run 3 Binding energy (kcal/mol)	Run 1 Binding energy (kcal/mol)	Run 2 Binding energy (kcal/mol)	Run 3 Binding energy (kcal/mol)
1	-4.56	-4.45	-4.18	-8.10	-8.37	-8.39
2	-4.50	-4.34	-4.24	-7.88	-6.90	-7.83
3	-4.12	-4.65	-4.09	-6.70	-6.57	-5.94
4	-4.23	-4.44	-4.53	-6.25	-6.69	-7.42
5	-4.78	-4.19	-4.20	-7.70	-6.39	-7.95
6	-4.33	-4.35	-4.63	-7.71	-6.65	-6.65
7	-4.96	-4.06	-4.74	-7.84	-7.81	-7.29
8	-4.30	-4.26	-5.04	-6.66	-7.87	-6.16
9	-4.20	-4.84	-4.19	-6.24	-8.15	-5.91

3. IL6_1ALU

	10-Hydroxygeraniol		
	Run 1 Binding energy (kcal/mol)	Run 2 Binding energy (kcal/mol)	Run 3 Binding energy (kcal/mol)
1	-4.15	-4.76	-4.61
2	-3.39	-4.75	-4.59
3	-4.22	-3.56	-3.72
4	-4.35	-3.46	-3.73
5	-4.48	-3.79	-4.17
6	-4.50	-3.23	-4.74
7	-4.07	-3.49	-4.77
8	-4.81	-3.22	-5.11
9	-3.18	-4.51	-3.35

4. STAT3_6NJS

	Benzyladenine			Caffeic acid			Rehmaglutin A		
	Run 1 Binding energy (kcal/mol)	Run 2 Binding energy (kcal/mol)	Run 3 Binding energy (kcal/mol)	Run 1 Binding energy (kcal/mol)	Run 2 Binding energy (kcal/mol)	Run 3 Binding energy (kcal/mol)	Run 1 Binding energy (kcal/mol)	Run 2 Binding energy (kcal/mol)	Run 3 Binding energy (kcal/mol)
1	-4.06	-4.13	-4.99	-4.24	-4.98	-4.56	-4.41	-4.31	-4.01
2	-4.36	-4.54	-5.31	-5.05	-5.11	-4.02	-4.19	-4.51	-3.92
3	-4.06	-4.01	-3.91	-4.66	-3.77	-5.06	-4.09	-4.29	-4.04
4	-4.85	-4.00	-4.31	-5.39	-4.68	-4.59	-4.51	-4.54	-4.32
5	-5.01	-4.02	-3.65	-4.63	-5.42	-5.12	-3.98	-4.33	-4.19
6	-4.20	-4.27	-4.95	-4.45	-3.81	-5.40	-4.54	-4.54	-4.33
7	-4.50	-4.85	-4.03	-4.15	-3.91	-4.57	-4.21	-4.50	-4.08
8	-4.38	-4.44	-5.09	-3.93	-4.51	-4.51	-4.34	-4.36	-4.80
9	-4.66	-4.99	-3.87	-5.60	-5.59	-3.99	-3.83	-4.04	-3.96

	Jioglutin C			Rehmaglutin D			Rehmaglutin B		
	Run 1 Binding energy (kcal/mol)	Run 2 Binding energy (kcal/mol)	Run 3 Binding energy (kcal/mol)	Run 1 Binding energy (kcal/mol)	Run 2 Binding energy (kcal/mol)	Run 3 Binding energy (kcal/mol)	Run 1 Binding energy (kcal/mol)	Run 2 Binding energy (kcal/mol)	Run 3 Binding energy (kcal/mol)
1	-4.87	-4.32	-5.62	-4.48	-3.96	-4.37	-4.29	-4.53	-4.23
2	-4.37	-5.63	-4.37	-5.28	-5.27	-4.15	-4.57	-4.57	-4.28
3	-4.23	-4.29	-4.38	-3.98	-4.10	-5.27	-4.40	-4.75	-4.25
4	-4.17	-4.24	-4.61	-4.14	-3.80	-4.10	-4.84	-4.64	-5.68
5	-3.57	-4.96	-4.57	-4.01	-4.15	-4.31	-5.70	-5.69	-4.65
6	-4.52	-4.31	-4.74	-4.99	-4.19	-4.99	-4.36	-4.68	-4.04
7	-4.57	-4.42	-4.92	-5.07	-4.03	-4.14	-4.68	-4.37	-4.12
8	-4.62	-4.38	-4.42	-3.94	-4.01	-4.44	-4.53	-5.43	-5.43
9	-4.62	-4.27	-4.54	-4.46	-4.99	-4.02	-4.51	-4.53	-4.97

	Rehmaglutin C			Rehmapicroside			Dihydrocatalpolgenin α		
	Run 1 Binding energy (kcal/mol)	Run 2 Binding energy (kcal/mol)	Run 3 Binding energy (kcal/mol)	Run 1 Binding energy (kcal/mol)	Run 2 Binding energy (kcal/mol)	Run 3 Binding energy (kcal/mol)	Run 1 Binding energy (kcal/mol)	Run 2 Binding energy (kcal/mol)	Run 3 Binding energy (kcal/mol)
1	-4.29	-3.81	-3.74	-5.35	-5.57	-5.79	-4.01	-5.05	-4.89
2	-3.94	-3.83	-4.27	-5.70	-5.59	-5.24	-4.49	-4.55	-4.29
3	-4.35	-3.81	-3.75	-5.51	-5.92	-6.05	-4.50	-4.01	-4.48
4	-4.58	-3.89	-4.07	-4.77	-6.37	-5.57	-4.02	-4.48	-4.52
5	-4.57	-4.38	-3.91	-5.07	-6.47	-6.23	-3.81	-4.26	-4.02
6	-4.58	-4.06	-3.96	-5.69	-5.54	-5.33	-3.71	-4.54	-4.11
7	-4.21	-3.85	-3.98	-4.86	-6.24	-6.36	-4.70	-4.25	-4.17
8	-4.15	-4.43	-4.46	-6.24	-5.88	-6.42	-4.73	-4.27	-4.72
9	-4.54	-5.01	-3.78	-4.47	-5.04	-5.51	-3.78	-4.42	-4.54

5. BCL2_2W3L

	Lauric acid			Palmitoleic acid			Tyrosol		
	Run 1 Binding energy (kcal/mol)	Run 2 Binding energy (kcal/mol)	Run 3 Binding energy (kcal/mol)	Run 1 Binding energy (kcal/mol)	Run 2 Binding energy (kcal/mol)	Run 3 Binding energy (kcal/mol)	Run 1 Binding energy (kcal/mol)	Run 2 Binding energy (kcal/mol)	Run 3 Binding energy (kcal/mol)
1	-4.05	-3.90	-4.65	-5.39	-4.65	-5.40	-3.88	-4.02	-3.79
2	-4.57	-4.64	-4.66	-4.47	-4.24	-4.75	-4.11	-4.17	-4.96
3	-3.99	-3.96	-3.95	-4.75	-4.99	-5.45	-4.16	-3.96	-4.41
4	-3.90	-4.21	-4.49	-5.28	-4.42	-5.37	-4.90	-4.96	-3.67
5	-4.25	-4.87	-4.60	-4.99	-4.88	-5.19	-4.13	-4.36	-4.55
6	-3.87	-4.71	-4.03	-4.17	-4.58	-4.49	-3.85	-3.75	-4.17
7	-4.70	-4.21	-3.89	-4.62	-4.70	-4.81	-4.07	-4.21	-4.44
8	-4.19	-4.33	-4.68	-4.03	-4.23	-4.11	-4.41	-4.27	-4.61
9	-4.45	-4.91	-4.43	-4.17	-4.07	-4.95	-4.18	-3.95	-3.82

6. TNF_2AZ5

	Iondiolone		
	Run 1 Binding energy (kcal/mol)	Run 2 Binding energy (kcal/mol)	Run 3 Binding energy (kcal/mol)
1	-6.60	-6.77	-6.12
2	-6.46	-6.76	-6.65
3	-6.78	-5.28	-6.77
4	-6.75	-7.22	-7.25
5	-5.89	-5.71	-6.76
6	-7.24	-7.22	-7.22
7	-5.51	-5.73	-5.73
8	-5.44	-5.48	-5.67
9	-7.22	-5.44	-5.50

7. NFKB1_8TQD

	Iridodial		
	Run 1 Binding energy (kcal/mol)	Run 2 Binding energy (kcal/mol)	Run 3 Binding energy (kcal/mol)
1	-3.55	-1.38	-3.16
2	-4.03	-3.56	-3.26
3	-4.00	-3.99	-2.99
4	-3.26	-3.77	-2.99
5	-3.26	-3.61	-3.28
6	-3.42	-3.95	-3.76
7	-3.38	-4.09	-3.78
8	-3.20	-4.44	-3.55
9	-3.41	-3.84	-3.84

8. PTGS2_5F19

	Caffeic acid			Phenylacetic acid			Geraniol		
	Run 1 Binding energy (kcal/mol)	Run 2 Binding energy (kcal/mol)	Run 3 Binding energy (kcal/mol)	Run 1 Binding energy (kcal/mol)	Run 2 Binding energy (kcal/mol)	Run 3 Binding energy (kcal/mol)	Run 1 Binding energy (kcal/mol)	Run 2 Binding energy (kcal/mol)	Run 3 Binding energy (kcal/mol)
1	-5.41	-5.47	-5.41	-4.06	-4.37	-4.21	-4.36	-5.17	-6.16
2	-7.34	-7.32	-7.29	-6.92	-4.03	-4.02	-3.93	-5.25	-4.91
3	-7.30	-7.29	-6.53	-4.25	-6.92	-4.27	-6.22	-4.15	-4.51
4	-5.96	-5.57	-6.53	-4.38	-4.35	-5.56	-4.03	-5.40	-5.99
5	-6.54	-7.14	-7.19	-5.33	-4.70	-6.92	-6.63	-5.57	-6.39
6	-5.84	-6.80	-5.24	-6.19	-4.18	-4.19	-4.44	-5.28	-5.17
7	-5.31	-5.90	-6.46	-4.02	-4.40	-4.43	-3.93	-4.08	-4.30
8	-6.41	-5.56	-5.46	-6.02	-5.83	-4.38	-4.05	-6.27	-4.44
9	-5.31	-7.19	-6.13	-5.80	-5.71	-5.49	-4.30	-4.12	-4.29

	Octanoic acid			Palmitoleic acid			5-Hydroxytryptamine		
	Run 1 Binding energy (kcal/mol)	Run 2 Binding energy (kcal/mol)	Run 3 Binding energy (kcal/mol)	Run 1 Binding energy (kcal/mol)	Run 2 Binding energy (kcal/mol)	Run 3 Binding energy (kcal/mol)	Run 1 Binding energy (kcal/mol)	Run 2 Binding energy (kcal/mol)	Run 3 Binding energy (kcal/mol)
1	-4.95	-3.61	-4.68	-5.38	-5.52	-4.71	-5.17	-5.20	-5.20
2	-3.39	-3.62	-5.26	-5.67	-4.75	-5.47	-6.28	-5.29	-4.99
3	-5.40	-4.01	-3.46	-5.45	-5.35	-4.73	-5.25	-4.95	-6.26
4	-3.31	-4.29	-4.56	-5.26	-5.59	-4.71	-5.18	-5.58	-4.81
5	-4.09	-4.71	-4.50	-5.44	-4.71	-4.65	-4.90	-6.10	-4.75
6	-3.54	-4.06	-5.09	-5.65	-5.11	-4.95	-5.27	-5.20	-5.04
7	-4.82	-5.28	-5.45	-5.10	-5.43	-4.70	-6.47	-5.74	-6.04
8	-4.53	-5.05	-3.98	-5.07	-5.33	-4.74	-5.15	-5.19	-6.39
9	-3.56	-4.44	-3.65	-4.72	-5.77	-4.89	-5.71	-5.38	-5.40

	Iridodial			5-Oxoproline			Jiofuran		
	Run 1 Binding energy (kcal/mol)	Run 2 Binding energy (kcal/mol)	Run 3 Binding energy (kcal/mol)	Run 1 Binding energy (kcal/mol)	Run 2 Binding energy (kcal/mol)	Run 3 Binding energy (kcal/mol)	Run 1 Binding energy (kcal/mol)	Run 2 Binding energy (kcal/mol)	Run 3 Binding energy (kcal/mol)
1	-5.58	-5.58	-5.65	-5.81	-5.81	-5.81	-4.71	-5.07	-4.95
2	-5.64	-5.64	-5.57	-5.63	-5.64	-5.63	-4.66	-5.25	-5.30
3	-5.43	-5.43	-4.86	-5.83	-4.33	-5.82	-5.53	-5.00	-5.96
4	-5.27	-4.78	-4.86	-6.09	-5.82	-6.09	-4.71	-5.62	-6.57
5	-5.00	-5.28	-4.88	-5.61	-6.09	-5.62	-4.67	-6.56	-4.98
6	-5.19	-4.75	-5.56	-3.79	-5.61	-5.69	-5.16	-5.37	-5.02
7	-5.15	-4.87	-5.16	-3.99	-4.76	-4.23	-5.04	-5.19	-5.08
8	-4.91	-5.21	-5.03	-5.70	-3.89	-3.86	-4.85	-5.11	-5.21
9	-5.14	-4.77	-4.90	-4.74	-4.27	-5.64	-6.57	-4.81	-5.34

	Jioglutolide			Ajugoside			Rehmaglutin A		
	Run 1 Binding energy (kcal/mol)	Run 2 Binding energy (kcal/mol)	Run 3 Binding energy (kcal/mol)	Run 1 Binding energy (kcal/mol)	Run 2 Binding energy (kcal/mol)	Run 3 Binding energy (kcal/mol)	Run 1 Binding energy (kcal/mol)	Run 2 Binding energy (kcal/mol)	Run 3 Binding energy (kcal/mol)
1	-7.56	-7.55	-7.55	-8.53	-8.65	-8.89	-6.19	-5.51	-5.8
2	-5.25	-5.51	-5.51	-8.35	-8.72	-7.90	-5.8	-5.67	-5.61
3	-5.03	-5.39	-5.53	-8.31	-8.79	-8.85	-5.66	-5.81	-5.91

4	-5.4	-7.31	-7.31	-8.81	-7.53	-8.72	-5.66	-5.46	-6.22
5	-7.31	-5.36	-6.65	-8.72	-8.15	-8.13	-5.99	-5.89	-6.54
6	-5.31	-5.31	-5.31	-8.70	-8.39	-7.60	-6.04	-5.99	-6.21
7	-5.01	-5.38	-5.56	-8.55	-8.20	-7.52	-6.19	-5.67	-6.20
8	-5.16	-5.89	-6.13	-7.73	-8.68	-7.99	-5.90	-5.64	-6.17
9	-6.5	-5.25	-5.52	-8.77	-8.32	-8.39	-6.52	-6.52	-5.36

	Jioglutin C			Rehmaglutin D			Rehmaglutin B		
	Run 1 Binding energy (kcal/mol)	Run 2 Binding energy (kcal/mol)	Run 3 Binding energy (kcal/mol)	Run 1 Binding energy (kcal/mol)	Run 2 Binding energy (kcal/mol)	Run 3 Binding energy (kcal/mol)	Run 1 Binding energy (kcal/mol)	Run 2 Binding energy (kcal/mol)	Run 3 Binding energy (kcal/mol)
1	-6.58	-6.10	-6.02	-6.11	-5.11	-6.10	-5.91	-5.99	-5.71
2	-6.53	-6.36	-5.99	-6.09	-5.37	-6.02	-5.46	-5.63	-6.01
3	-5.94	-6.43	-6.73	-5.32	-5.69	-5.86	-5.51	-6.09	-5.46
4	-6.56	-6.24	-6.57	-5.45	-5.49	-5.41	-5.14	-5.74	-6.08
5	-6.17	-5.95	-6.04	-5.42	-5.32	-5.48	-5.29	-5.59	-5.76
6	-6.45	-6.56	-6.14	-5.38	-5.29	-5.46	-5.41	-6.56	-5.06
7	-5.97	-6.59	-6.12	-5.18	-5.24	-5.66	-5.74	-5.66	-5.74
8	-6.33	-5.79	-5.97	-6.16	-5.51	-5.44	-5.30	-6.28	-6.62
9	-6.13	-6.00	-6.10	-4.97	-5.4	-5.33	-6.62	-5.71	-5.18

	Jioglutin A			Jioglutin B			Rehmaglutin C		
	Run 1 Binding energy (kcal/mol)	Run 2 Binding energy (kcal/mol)	Run 3 Binding energy (kcal/mol)	Run 1 Binding energy (kcal/mol)	Run 2 Binding energy (kcal/mol)	Run 3 Binding energy (kcal/mol)	Run 1 Binding energy (kcal/mol)	Run 2 Binding energy (kcal/mol)	Run 3 Binding energy (kcal/mol)
1	-5.91	-6.37	-6.36	-5.58	-5.32	-5.66	-5.18	-5.28	-5.31
2	-6.25	-5.76	-5.57	-5.33	-5.95	-5.66	-5.29	-5.06	-5.89
3	-6.38	-5.97	-6.03	-5.08	-5.98	-5.67	-5.12	-6.61	-5.43
4	-5.77	-6.19	-5.51	-5.65	-6.58	-6.14	-5.23	-5.00	-5.37
5	-5.78	-6.06	-6.18	-5.76	-6.11	-6.68	-5.29	-5.34	-5.32
6	-5.84	-6.14	-5.97	-6.78	-5.55	-5.94	-5.07	-5.37	-5.28
7	-6.01	-5.74	-5.50	-6.07	-5.48	-5.79	-5.34	-5.41	-5.21
8	-5.78	-6.14	-5.53	-5.43	-5.84	-5.68	-5.40	-5.49	-5.36
9	-5.95	-5.76	-5.55	-5.62	-5.80	-5.76	-5.24	-5.03	-5.35

	Tyrosol			Hydroxymethyl furaldehyde			5-Methoxytryptamine		
	Run 1 Binding energy (kcal/mol)	Run 2 Binding energy (kcal/mol)	Run 3 Binding energy (kcal/mol)	Run 1 Binding energy (kcal/mol)	Run 2 Binding energy (kcal/mol)	Run 3 Binding energy (kcal/mol)	Run 1 Binding energy (kcal/mol)	Run 2 Binding energy (kcal/mol)	Run 3 Binding energy (kcal/mol)
1	-5.70	-5.76	-5.70	-4.71	-3.82	-4.72	-5.22	-4.99	-5.22
2	-6.44	-5.52	-5.67	-4.27	-5.08	-5.15	-5.00	-4.87	-5.18
3	-4.25	-5.81	-5.61	-4.05	-4.05	-3.76	-5.97	-5.98	-4.90
4	-5.94	-6.45	-6.46	-5.08	-4.89	-4.90	-4.91	-6.23	-4.91
5	-4.27	-4.37	-5.75	-4.78	-5.39	-3.48	-5.17	-4.95	-6.04
6	-4.17	-5.75	-5.61	-3.76	-3.79	-4.03	-5.95	-4.92	-6.12
7	-5.67	-4.46	-5.71	-3.73	-3.77	-4.88	-5.24	-4.97	-5.72
8	-4.51	-5.41	-4.45	-4.60	-4.13	-4.77	-4.87	-4.68	-4.77
9	-4.56	-5.61	-5.94	-4.69	-5.31	-3.57	-4.96	-5.42	-5.01

	Melatonin			10-Hydroxygeraniol			Jioglutin A diacetate		
	Run 1 Binding energy (kcal/mol)	Run 2 Binding energy (kcal/mol)	Run 3 Binding energy (kcal/mol)	Run 1 Binding energy (kcal/mol)	Run 2 Binding energy (kcal/mol)	Run 3 Binding energy (kcal/mol)	Run 1 Binding energy (kcal/mol)	Run 2 Binding energy (kcal/mol)	Run 3 Binding energy (kcal/mol)
1	-6.55	-6.67	-6.59	-6.68	-5.57	-4.57	-6.83	-5.60	-6.69
2	-6.64	-6.9	-6.96	-5.29	-4.74	-5.36	-6.27	-5.39	-6.01
3	-5.89	-7.01	-6.89	-4.20	-4.31	-4.71	-6.45	-6.84	-6.86
4	-6.97	-6.35	-6.31	-4.87	-4.54	-5.37	-5.83	-6.89	-6.63
5	-6.38	-6.95	-6.67	-5.45	-5.41	-6.37	-7.23	-6.78	-6.89
6	-7.02	-5.92	-5.98	-4.36	-6.61	-4.32	-5.48	-5.55	-6.97
7	-5.51	-5.9	-5.55	-4.80	-5.39	-5.29	-6.95	-5.83	-6.64
8	-5.85	-6.15	-5.72	-4.38	-4.56	-5.24	-6.76	-6.67	-6.05
9	-6.1	-6.05	-6.18	-4.20	-5.80	-4.18	-6.89	-7.03	-6.90

	Dihydrocatalpolgenin α			Methyl-pyrrole-carbaldehyde			1-Naphthaleneacetic acid		
	Run 1 Binding energy (kcal/mol)	Run 2 Binding energy (kcal/mol)	Run 3 Binding energy (kcal/mol)	Run 1 Binding energy (kcal/mol)	Run 2 Binding energy (kcal/mol)	Run 3 Binding energy (kcal/mol)	Run 1 Binding energy (kcal/mol)	Run 2 Binding energy (kcal/mol)	Run 3 Binding energy (kcal/mol)
1	-5.54	-5.33	-5.34	-5.01	-5.11	-5.10	-6.20	-6.08	-6.54
2	-5.44	-5.54	-5.54	-5.12	-5.09	-5.04	-5.33	-5.84	-5.78
3	-5.40	-5.54	-5.55	-3.71	-3.87	-4.24	-6.10	-6.59	-6.12
4	-5.23	-5.46	-5.84	-3.69	-3.65	-3.58	-6.48	-5.88	-5.88
5	-5.78	-5.52	-5.76	-3.72	-4.41	-3.49	-5.58	-5.96	-5.92
6	-5.25	-5.30	-5.40	-3.75	-3.91	-3.55	-5.75	-5.55	-5.67
7	-5.52	-5.38	-5.42	-4.26	-5.18	-4.66	-5.44	-6.13	-6.83
8	-5.74	-5.74	-5.67	-3.79	-5.59	-3.61	-5.64	-5.89	-5.45
9	-5.26	-5.29	-5.27	-5.58	-3.68	-3.97	-5.90	-6.57	-5.56

9. IL1B_3LTQ

	Rehmaglutin C		
	Run 1 Binding energy (kcal/mol)	Run 2 Binding energy (kcal/mol)	Run 3 Binding energy (kcal/mol)
1	-4.60	-4.29	-4.17
2	-3.74	-3.55	-3.59
3	-4.13	-3.78	-3.96
4	-4.17	-3.35	-3.69
5	-4.74	-4.03	-4.22
6	-3.84	-3.52	-3.87
7	-3.84	-3.60	-4.68
8	-4.08	-3.72	-4.51
9	-3.78	-4.65	-4.11

10. CASP3_3EDQ

	Geranyl diphosphate			Deoxyloganic acid			Jiofuran		
	Run 1 Binding energy (kcal/mol)	Run 2 Binding energy (kcal/mol)	Run 3 Binding energy (kcal/mol)	Run 1 Binding energy (kcal/mol)	Run 2 Binding energy (kcal/mol)	Run 3 Binding energy (kcal/mol)	Run 1 Binding energy (kcal/mol)	Run 2 Binding energy (kcal/mol)	Run 3 Binding energy (kcal/mol)
1	-4.66	-4.27	-3.85	-5.15	-5.97	-5.57	-5.24	-5.25	-5.24
2	-4.39	-4.65	-4.27	-5.54	-6.37	-6.01	-4.93	-4.01	-3.87
3	-4.87	-4.33	-4.01	-5.54	-5.18	-5.47	-4.22	-4.43	-3.71
4	-4.06	-4.14	-3.82	-6.87	-5.19	-5.17	-4.51	-3.89	-4.94
5	-4.87	-4.84	-5.87	-5.65	-6.60	-6.77	-3.70	-3.83	-3.99
6	-4.03	-4.57	-5.11	-5.56	-5.34	-5.69	-3.66	-3.83	-3.80
7	-4.30	-4.49	-3.83	-7.75	-7.06	-6.03	-3.91	-4.41	-3.76
8	-4.63	-4.61	-5.33	-5.21	-5.44	-7.70	-4.42	-3.79	-4.18
9	-4.21	-4.16	-4.02	-5.83	-7.69	-5.59	-4.28	-3.95	-4.49

	Jioglutolide			Rehmaglutin A			Jioglutin C		
	Run 1 Binding energy (kcal/mol)	Run 2 Binding energy (kcal/mol)	Run 3 Binding energy (kcal/mol)	Run 1 Binding energy (kcal/mol)	Run 2 Binding energy (kcal/mol)	Run 3 Binding energy (kcal/mol)	Run 1 Binding energy (kcal/mol)	Run 2 Binding energy (kcal/mol)	Run 3 Binding energy (kcal/mol)
1	-3.57	-4.17	-3.59	-4.67	-5.23	-5.24	-4.23	-5.46	-5.04
2	-3.51	-5.08	-5.02	-5.11	-4.67	-4.19	-4.30	-4.33	-5.59
3	-3.65	-5.25	-5.26	-5.39	-4.26	-4.52	-5.45	-4.77	-4.61
4	-5.37	-3.50	-3.86	-4.26	-5.37	-3.98	-5.60	-4.55	-4.81
5	-5.26	-4.24	-4.34	-4.48	-4.94	-3.97	-5.48	-4.23	-5.46
6	-4.03	-3.76	-4.17	-4.95	-4.71	-3.86	-4.31	-4.06	-4.43
7	-3.32	-3.50	-3.65	-4.80	-4.01	-4.13	-4.57	-4.45	-5.98
8	-3.61	-4.37	-3.82	-4.09	-4.20	-3.75	-4.31	-4.68	-4.76
9	-4.28	-4.06	-4.43	-5.09	-4.03	-4.21	-4.80	-4.23	-4.60

	Rehmaglutin D			Rehmaglutin B			Jioglutin A		
	Run 1 Binding energy (kcal/mol)	Run 2 Binding energy (kcal/mol)	Run 3 Binding energy (kcal/mol)	Run 1 Binding energy (kcal/mol)	Run 2 Binding energy (kcal/mol)	Run 3 Binding energy (kcal/mol)	Run 1 Binding energy (kcal/mol)	Run 2 Binding energy (kcal/mol)	Run 3 Binding energy (kcal/mol)
1	-4.81	-5.08	-4.86	-5.03	-4.18	-4.03	-4.37	-3.85	-5.54
2	-5.23	-4.72	-5.24	-4.20	-4.03	-4.06	-5.54	-5.53	-5.51
3	-4.37	-4.29	-4.07	-5.44	-4.41	-5.44	-5.34	-3.86	-4.43
4	-4.96	-4.37	-4.26	-4.27	-5.57	-4.47	-3.98	-3.55	-4.09
5	-3.58	-4.24	-4.30	-5.56	-4.04	-5.08	-3.99	-4.20	-4.05
6	-3.70	-4.08	-4.63	-5.19	-5.16	-4.54	-3.91	-4.04	-4.18
7	-3.56	-3.91	-4.48	-4.65	-4.20	-4.34	-4.17	-4.16	-4.61
8	-3.80	-4.14	-5.01	-4.48	-4.51	-4.94	-4.30	-4.74	-4.89
9	-3.93	-4.17	-3.96	-4.55	-5.29	-4.37	-4.67	-5.04	-4.96

	Jioglutin B			Rehmapicroside			Uridine		
	Run 1 Binding energy (kcal/mol)	Run 2 Binding energy (kcal/mol)	Run 3 Binding energy (kcal/mol)	Run 1 Binding energy (kcal/mol)	Run 2 Binding energy (kcal/mol)	Run 3 Binding energy (kcal/mol)	Run 1 Binding energy (kcal/mol)	Run 2 Binding energy (kcal/mol)	Run 3 Binding energy (kcal/mol)
1	-4.02	-3.92	-3.84	-5.44	-5.3	-5.17	-4.47	-5.42	-5.38
2	-5.49	-4.04	-3.71	-6.72	-5.33	-6.80	-4.57	-4.16	-4.84
3	-4.43	-4.82	-5.12	-5.33	-5.08	-5.30	-4.43	-4.25	-4.47
4	-4.23	-4.47	-3.95	-5.6	-6.83	-5.99	-4.52	-4.5	-4.26
5	-4.4	-5.51	-3.94	-5.46	-6.24	-5.38	-5.04	-4.77	-4.86
6	-4.21	-4.28	-3.81	-6.70	-6.14	-5.43	-5.79	-4.21	-5.14
7	-4.78	-5.17	-5.39	-5.24	-6.47	-5.02	-5.60	-4.41	-4.31
8	-4.43	-3.92	-4.43	-5.71	-6.24	-5.33	-6.05	-5.59	-4.30
9	-4.31	-4.52	-3.98	-6.89	-6.10	-5.22	-5.06	-4.10	-4.79

	Tyrosol			DMAPP			IPP		
	Run 1 Binding energy (kcal/mol)	Run 2 Binding energy (kcal/mol)	Run 3 Binding energy (kcal/mol)	Run 1 Binding energy (kcal/mol)	Run 2 Binding energy (kcal/mol)	Run 3 Binding energy (kcal/mol)	Run 1 Binding energy (kcal/mol)	Run 2 Binding energy (kcal/mol)	Run 3 Binding energy (kcal/mol)
1	-3.83	-3.32	-3.43	-3.66	-3.53	-3.83	-4.60	-4.91	-3.5
2	-3.95	-3.23	-3.21	-4.04	-3.38	-3.65	-4.31	-4.81	-4.85
3	-3.64	-3.69	-3.13	-3.68	-3.82	-3.58	-3.85	-3.94	-4.02
4	-3.81	-3.11	-3.36	-3.65	-4.20	-3.45	-3.82	-3.76	-3.73
5	-3.04	-3.17	-3.17	-4.1	-3.91	-3.72	-4.20	-3.57	-3.52
6	-3.46	-3.07	-3.25	-3.82	-3.46	-3.37	-3.85	-3.72	-3.42
7	-3.19	-3.35	-3.72	-3.63	-4.96	-3.92	-4.86	-4.69	-3.43
8	-3.44	-3.16	-3.06	-3.44	-3.85	-4.30	-4.06	-4.01	-3.85
9	-3.56	-3.28	-5.07	-3.69	-4.85	-4.03	-4.40	-3.86	-4.27

	Jioglutin A diacetate			Dihydrocatalpolgenin α			Methyl-pyrrole-carbaldehyde		
	Run 1 Binding energy (kcal/mol)	Run 2 Binding energy (kcal/mol)	Run 3 Binding energy (kcal/mol)	Run 1 Binding energy (kcal/mol)	Run 2 Binding energy (kcal/mol)	Run 3 Binding energy (kcal/mol)	Run 1 Binding energy (kcal/mol)	Run 2 Binding energy (kcal/mol)	Run 3 Binding energy (kcal/mol)
1	-2.43	-5.53	-5.26	-4.11	-4.3	-5.29	-3.13	-3.00	-2.95
2	-5.53	-5.53	-5.54	-5.18	-5.25	-4.11	-4.47	-3.05	-3.03
3	-4.91	-4.62	-1.97	-5.00	-4.42	-5.38	-2.86	-4.46	-3.08
4	-4.70	-5.31	-5.25	-4.10	-4.34	-4.22	-2.75	-3.12	-2.99
5	-5.25	-4.14	-4.42	-4.23	-4.12	-4.70	-2.93	-3.26	-3.00
6	-4.28	-4.34	-4.39	-4.23	-4.64	-4.49	-4.55	-3.01	-4.46
7	-4.82	-4.22	-4.03	-4.10	-5.38	-5.05	-2.87	-4.55	-3.37
8	-5.36	-4.67	-4.24	-5.38	-4.65	-5.00	-2.90	-3.45	-3.44
9	-4.28	-4.17	-4.26	-4.41	-4.33	-4.31	-2.73	-3.01	-4.53

11.GAPDH_6IQ6

	Ajugoside			Jioglutin C			Jioglutin A		
	Run 1 Binding energy (kcal/mol)	Run 2 Binding energy (kcal/mol)	Run 3 Binding energy (kcal/mol)	Run 1 Binding energy (kcal/mol)	Run 2 Binding energy (kcal/mol)	Run 3 Binding energy (kcal/mol)	Run 1 Binding energy (kcal/mol)	Run 2 Binding energy (kcal/mol)	Run 3 Binding energy (kcal/mol)
1	-9.33	-8.25	-7.70	-6.90	-6.87	-7.06	-7.25	-6.88	-7.24
2	-9.53	-9.72	-8.15	-7.12	-7.17	-7.10	-7.65	-6.34	-7.65
3	-9.83	-7.75	-10.3	-7.95	-7.89	-7.95	-7.85	-6.40	-7.44
4	-10.15	-10.17	-8.05	-7.89	-6.55	-7.80	-7.84	-7.64	-7.86
5	-7.94	-9.16	-9.34	-6.57	-7.95	-6.78	-7.45	-7.27	-7.84
6	-9.29	-8.69	-9.38	-7.07	-6.76	-7.01	-7.43	-7.86	-7.05
7	-8.73	-10.35	-8.12	-6.70	-6.68	-7.13	-6.99	-7.86	-6.57
8	-8.07	-8.49	-9.26	-7.64	-6.67	-6.70	-7.53	-6.97	-7.24
9	-9.39	-9.04	-9.97	-7.56	-7.44	-7.09	-6.9	-6.37	-7.26

	Jioglutin B			Tachioside			Isotachioside		
	Run 1 Binding energy (kcal/mol)	Run 2 Binding energy (kcal/mol)	Run 3 Binding energy (kcal/mol)	Run 1 Binding energy (kcal/mol)	Run 2 Binding energy (kcal/mol)	Run 3 Binding energy (kcal/mol)	Run 1 Binding energy (kcal/mol)	Run 2 Binding energy (kcal/mol)	Run 3 Binding energy (kcal/mol)
1	-7.25	-6.88	-7.24	-8.74	-8.6	-7.96	-8.67	-8.50	-8.19
2	-7.65	-6.34	-7.65	-7.91	-7.82	-7.72	-8.88	-8.47	-8.78
3	-7.85	-6.40	-7.44	-7.95	-8.09	-8.61	-8.15	-8.05	-7.31
4	-7.84	-7.64	-7.86	-8.07	-7.97	-8.64	-8.32	-8.24	-8.14
5	-7.45	-7.27	-7.84	-8.40	-8.53	-8.22	-7.97	-8.90	-8.17
6	-7.43	-7.86	-7.05	-8.31	-8.24	-8.26	-8.44	-8.46	-7.69
7	-6.99	-7.86	-6.57	-8.11	-8.50	-8.82	-7.95	-8.74	-8.11
8	-7.53	-6.97	-7.24	-8.38	-9.41	-7.56	-8.54	-8.33	-8.36
9	-6.90	-6.37	-7.26	-9.30	-8.09	-9.41	-8.40	-9.06	-8.41

	Uridine			Rhodioloside			Oxireno cyclopenta pyrandiol		
	Run 1 Binding energy (kcal/mol)	Run 2 Binding energy (kcal/mol)	Run 3 Binding energy (kcal/mol)	Run 1 Binding energy (kcal/mol)	Run 2 Binding energy (kcal/mol)	Run 3 Binding energy (kcal/mol)	Run 1 Binding energy (kcal/mol)	Run 2 Binding energy (kcal/mol)	Run 3 Binding energy (kcal/mol)
1	-7.35	-6.70	-7.58	-8.52	-7.68	-7.56	-6.24	-5.96	-6.69
2	-7.14	-6.35	-7.38	-9.17	-7.41	-7.43	-5.94	-6.01	-6.64
3	-7.56	-7.56	-7.47	-7.66	-7.76	-7.58	-6.60	-6.10	-6.50
4	-7.62	-6.50	-7.72	-7.41	-7.70	-8.75	-6.83	-6.73	-6.70
5	-7.39	-7.59	-7.15	-9.14	-9.14	-7.57	-6.08	-6.8	-6.27
6	-7.69	-6.22	-7.69	-7.13	-7.71	-7.26	-6.80	-6.92	-7.67
7	-7.15	-6.35	-6.48	-7.68	-8.79	-7.60	-6.69	-6.08	-6.86
8	-7.25	-7.48	-6.60	-7.81	-7.6	-7.66	-6.10	-6.63	-7.65
9	-7.51	-6.58	-6.91	-7.4	-7.74	-9.23	-7.65	-7.64	-6.79
